# A new perspective on the regulation of glucose and cholesterol transport by mitochondria-lysosome contact sites

**DOI:** 10.3389/fphys.2024.1431030

**Published:** 2024-09-03

**Authors:** Xiaolong Chen, Chun Guang Li, Xian Zhou, Minghua Zhu, Jing Jin, Ping Wang

**Affiliations:** ^1^ School of Physical Education, Hangzhou Normal University, Hangzhou, China; ^2^ NICM Health Research Institute, Western Sydney University, Westmead, NSW, Australia; ^3^ Department of Cardiothoracic Surgery, Affiliated Hospital of Hangzhou Normal University, Hangzhou, China

**Keywords:** mitochondria-lysosome contact sites, glucose transport, cholesterol transport, insulin resistance, nonalcoholic fatty liver disease

## Abstract

Mitochondria and lysosomes play a very important role in maintaining cellular homeostasis, and the dysfunction of these organelles is closely related to many diseases. Recent studies have revealed direct interactions between mitochondria and lysosomes, forming mitochondria-lysosome contact sites that regulate organelle network dynamics and mediate the transport of metabolites between them. Impaired function of these contact sites is not only linked to physiological processes such as glucose and cholesterol transport but also closely related to the pathological processes of metabolic diseases. Here, we highlight the recent progress in understanding the mitochondria-lysosome contact sites, elucidate their role in regulating metabolic homeostasis, and explore the potential implications of this pathway in metabolic disorders.

## 1 Introduction

The obesity epidemic is increasing dramatically around the world, and the number of affected individuals continue to rise (Jin et al., 2023). The World Health Organization (WHO) predicts that it will increase by approximately 167 million people by 2025 (Jin et al., 2023). The reasons may be related to changes in diet (Janssen et al., 2004), decrease in physical activity (Church et al., 2011), and increase in sedentary behaviour (Sugiyama et al., 2008). As obesity rates increase, so do the incidence and prevalence of insulin resistance (IR), nonalcoholic fatty liver disease (NAFLD), and other metabolic diseases. These conditions are risk factors for type 2 diabetes mellitus (T2DM) and cardiovascular and cerebrovascular diseases (Kivimäki et al., 2017). Therefore, it is particularly important to study the molecular mechanisms of obesity-induced IR, NAFDL and other metabolic diseases.

Among the many mechanisms in the occurrence and development of IR and NAFLD induced by obesity, recent attention has been drawn to how different organelles in the cell communicate with each other. Among these, the mechanism of mitochondrial and lysosome interdependence and interactions is the current research hotspot. The direct crosstalk at mitochondria and lysosome contact sites formed by specific proteins participate in signal transduction and metabolite transfer between organelles has recently been recognized as an important mechanism for maintaining the stability of cellular function (Simmen and Tagaya, 2017). This mechanism has lately been extensively studied in metabolic diseases, and the mitochondria and lysosome contact sites have been found to be related to the exchanges of various metabolites such as calcium, cholesterol, iron (Gatta and Levine, 2017) as well as to regulation of mitochondrial dynamics (Wong et al., 2018). Impairment of mitochondria-lysosome membrane contact sites may result in dyslipidemia and the progressive accumulation of intramyocellular fat content, reducing glucose transport and promoting cholesterol aggregation, leading to cytolipotoxicity and decreased insulin sensitivity, contributing to IR and NAFLD (Lin et al., 2023; Progida et al., 2010). In addition, damaged mitochondria-lysosome contact sites have also been shown to correlate with acute cardiac injury and neurodegenerative diseases (Cisneros et al., 2022; Yu et al., 2020). This review focuses on a synopsis of mitochondria-lysosome membrane contact sites, highlighting recent results on the regulation and mechanism of mitochondria-lysosome contact sites in glycolipid metabolism homeostasis, and discuss their roles in pathogenesis of the pathology of metabolic diseases.

## 2 Overview of mitochondrial and lysosomal interactions and contact sites

Mitochondria were discovered in the 1890s, and their role in cell metabolism was recognized in the 1920s and 1930s. Since Lynn Margulis (then Lynn Sagan) published the famous *On the Origin of Mitosing Cells* in 1967, it has been proposed that eukaryotic organelles, including mitochondria and chloroplasts, evolved from endosymbiotic bacteria (Sagan, 1967). Although the nature of the initial endosymbiosis is still uncertain, the generally accepted view is that mitochondria are the primary sites of cellular metabolism (Spinelli and Haigis, 2018). Lysosomes, on the other hand, were discovered by Nobel laureate de Duve C in 1955 (de Duve, 2005), and their role in cell degradation was recognized in the late 1950s and early 1960s. The generally accepted view is that they are the central organelles for cellular degradation (Holland et al., 2020). Both organelles play different biological functions. However, with the gradual deepening of research in recent years, scientists have realized that the functions of mitochondria and lysosomes are not independent, but interact with each other. The initial evidence came from findings by Rustom A et al. published in *Science* Rustom et al. (2004). Since then, various supporting evidence was obtained, including Fernandez-Mosquera et al. reported that when the respiratory chain of mitochondria has chronic defects, lysosomal biogenesis cannot be triggered even if the cells are starved of amino acids or inhibited by mTORC1, indicating that mitochondrial dysfunction affects the biogenesis and function of lysosomes (Fernandez-Mosquera et al., 2019). Conversely, it was observed that excessive accumulation of mitochondria Ca^2+^ led to mitochondrial rupture and a decline in mitochondrial function in Lysosomal storage-diseases (LSDs) (Cantarero et al., 2021), suggesting that mitochondrial biogenesis can be inhibited as a protective mechanism in cells and tissues when lysosomes are defective (Khalil et al., 2017), indicating that lysosomes and mitochondria are interdependent and coordinate across time and space barriers, to complete complex cell metabolism, signal transduction, and other functions to maintain the homeostasis of the whole cell (Qing-long, 2022).

As reported by Wu et al. (2018) in *Science*, the endoplasmic reticulum forms many contact sites with other parts of the cell, including mitochondria. The main role of these contact sites is to constantly transport lipids and small molecules between the endoplasmic reticulum and the other organelles such as mitochondria, which is important but not necessary. The direct evidence for mitochondria and lysosomes contact sites came from studies by Elbaz-Alon et al. (2014) who found contact sites between the lysosomal chamber (vacuole) and the mitochondria (named vCLAMP, vacuole and mitochondrial patch) in yeast, and vCLAMP is enriched with ion and amino acid transporters that play a function in lipid relay between the endomembrane system and mitochondria. Han Y later obtained dual-color structured illumination microscopy (SIM) images of dynamic physical interactions of lysosomal-mitochondria in human osteosarcoma cells by using the cell-permeable organic fluorescence probes (Han et al., 2017). Wong YC also reported in Nature the identification of mitochondrial and lysosome contact sites in mammalian cells using electron microscopy (Wong et al., 2018). Moreover, it has been shown that the average distance between mitochondria and lysosome contact sites is about 10 nm between mitochondrial and lysosomal membranes (Aston et al., 2017). These findings suggest that direct crosstalk dependent on mitochondria and lysosome contact sites may be an important mechanism to regulate cellular homeostasis.

**FIGURE 1:**
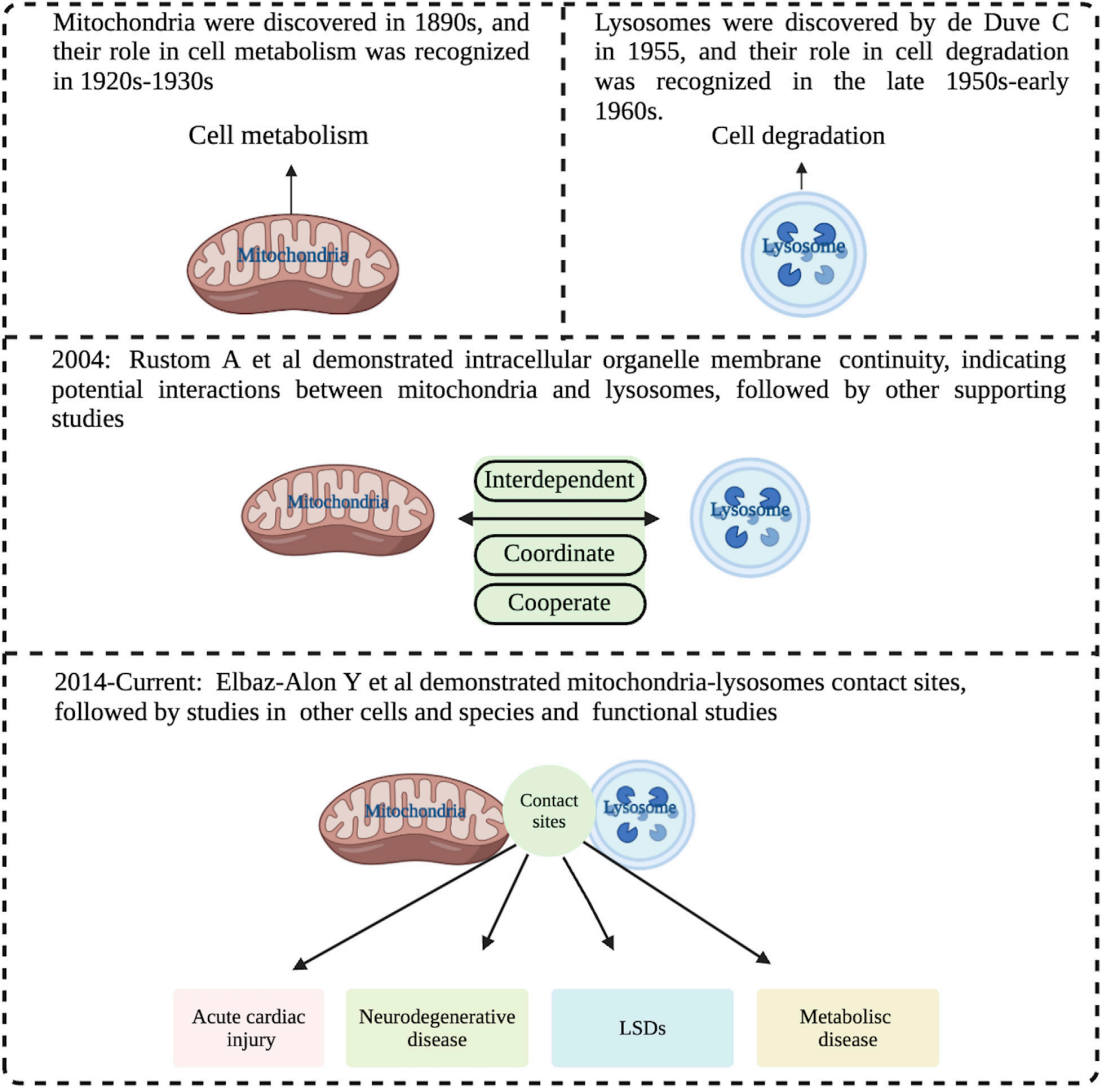
Historical view of the interaction between mitochondria and lysosomes. The early view was that mitochondria and lysosomes were independently responsible for cell metabolism and cell degradation respectively. The more recent view is that they are interdependent and coordinately to perform and regulate cell metabolism and signal transductions. The current view is that mitochondria and lysosomes form dynamic membrane contact sites, and abnormalities of these contact sites are associated with various diseases such as acute myocardial injury, neurodegenerative disease, LSDs and metabolism disease. LSDs: Lysosomal storage-disease. Image created with BioRender.com.

However, it was also observed that no significant transorganellar transfer such as lysosomal contents, mitochondrial matrix proteins, or membrane gap proteins occurred at the mitochondria-lysosome contact site (Wong et al., 2018). In addition, neither autophagosome biogenesis events nor mitophagy could occur at mitochondria-lysosome contact sites, as no markers from the phagosomes, including ULK1, Atg5, Atg12, and LC3 were detected (Wong et al., 2018). Mitochondria and lysosome contact sites also are not sites for lysosomal engulfment of bulk mitochondria (Wong et al., 2019). Importantly, dysfunction of mitochondria and lysosome contact sites has been linked to various human diseases. For example, flaws in mitochondria-lysosome contact sites have been discovered in genetic mutation associated with heart damage caused by acute myocardial infarction (Yu et al., 2020). Meanwhile, in many neurodegenerative diseases, such as Charcot-Marie-Tooth (CMT) disease, Alzheimer’s disease (AD), Amyotrophic lateral sclerosis (ALS) and Parkinson’s disease (PD), a common pathological basis is damaged dynamics and function of mitochondria-lysosome contact sites induced by the accumulation of mutant proteins in neurons (Cisneros et al., 2022; Poewe et al., 2017; Schiavon et al., 2021; Wong et al., 2018). Additionally, some LSDs, such as Niemann-Pick disease type C and neuronal ceroid lipofuscinosis, may be partly due to mitochondria-lysosome contact site dysfunction caused by lysosomal defects (Plotegher and Duchen, 2017) (Figure 1).

Recently, mitochondria-lysosome contact sites have been well-studied for their direct regulation of glucose and lipid metabolism and other metabolic diseases in healthy or abnormal mammalian cells in normal or stressed environments (Henne, 2019; Wu et al., 2019). The following mainly elaborates on the metabolism of glucose and lipids.

## 3 Dynamic process of tethering and untethering at mitochondria-lysosome contact sites

**FIGURE 2:**
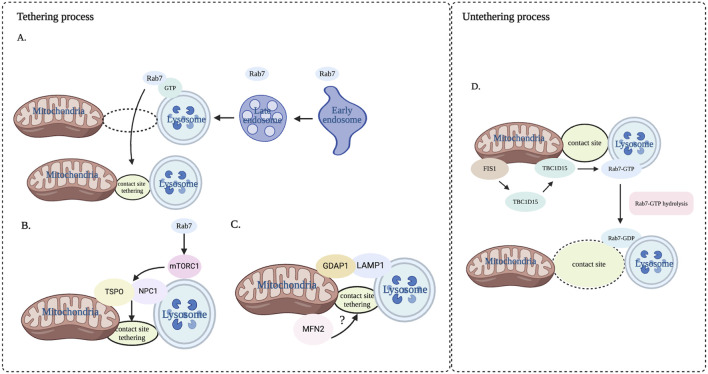
Dynamic process of tethering and untethering at mitochondria-lysosome contact sites. This dynamic process is regulated by a variety of proteins that form the mitochondrial and lysosomal membranes. **(A)** Rab7 in the late endosome accesses the contact sites. **(B)** Rab7 regulates tethering by facilitating the interaction of TSPO and NPC1 through mTOR1. **(C)** GDAP1 interacting with LAMP1 enhances the tethering of mitochondria-lysosome contact site, and MFN2 is also involved in the regulation of mitochondria-lysosome contact sites. **(D)** Untethering process primarily regulated by Rab7 GTP hydrolysis driven by TBC1D15. NPC1: Niemann-Pick type C1. TSPO: translocator protein. MFN2: protein mitofusin. Image created with BioRender.com.

Mitochondria-lysosome contact sites are dynamic and variable, involving two processes of tethering and untethering, primarily mediated by the interaction of various proteins present on the mitochondrial membranes and lysosomal membranes (Wong et al., 2019) (Figure 2). Among these, Rab7, a small G protein in the Rab family, plays an essential role in regulating the maturation from early endosome to late endosome and the transport and fusion from late endosome to lysosome (Progida et al., 2010). There is evidence that Rab7 can modulate the dynamics of tethering and untethering at mitochondria-lysosome contact sites by its alternating between lysosomal-localized GTP-binding state (an active) and cytosolic GDP-binding state (an inactive) (Wong et al., 2019). Interestingly, the expression of the constitutively active GTP-bound Rab7 (Q67L) mutant, which cannot undergo GTP hydrolysis, give rise to an increased percentage of mitochondrial-lysosomal contact sites and prolonging the contact duration compared to the wild-type Rab7 (Wong et al., 2018). It is worth noting that the lysosomal mammalian target of rapamycin complex 1 (mTORC1) is located downstream of Rab7 and the central role of mTORC1 is lysosomal signal transduction (Düvel et al., 2010). Lin et al. found that mTORC1 promoted the formation of mitochondria-lysosome contact sites tethering and mediated cholesterol transport from lysosome to mitochondria by regulating the interaction between NPC1 and the mitochondrial translocation protein (TSPO) (Lin et al., 2023). We speculate that the complex that is required for the mitochondria-lysosome contact sites formation may be composed of Rab7 and its effector protein, providing a platform for modulating metabolite flux between organelles.

In addition, other protein complexes have been also discovered as regulators of mitochondria-lysosome contact sites tethering. One such protein is GDAP1, which includes two Glutathione S-transferase (GST) domains. GDAP1 is a complete membrane protein on the outer membrane of mitochondria (Huber et al., 2013). Studies have shown that GDAP1 interacted with LAMP1 protein on the lysosomal membrane at the mitochondria-lysosome contact site, and this interaction enhanced the affinity between mitochondria and lysosomes (Audano et al., 2018). Knockdown of GDAP1 increased the distance between mitochondria and lysosomes and decreased the amount and duration of mitochondria-lysosome contact sites (Audano et al., 2018). Additionally, mitochondrial fusion protein 2 (MFN2) protein located primarily in the mitochondrial outer membrane is also jointly regulating mitochondria-lysosome contact sites. Knockdown of MFN2 leads to a decreased number of mitochondria-lysosome contact sites in primary human erythroid progenitors, suggesting the involvement of MFN2 in the regulation of these contact sites (Li et al., 2022). We speculate that the complex that promotes the formation of mitochondrial lysosome contact sites may involve other proteins in addition to those mentioned above, which is not clear at present and needs further research.

Another dynamic process at mitochondria-lysosome contact sites is the untethering, primarily regulated by the hydrolysis of Rab7 GTP. The hydrolysis of Rab7 GTP at mitochondria-lysosome contact sites is driven by TBC1D15, which is a kind of Rab7 GTPase activating protein (GAP) and is recruited to the mitochondria *via* the mitochondrial extramembrane protein Fis1. Once recruited to the mitochondria, TBC1D15 interacts with lysosomal GTP-bound Rab7 at the contact site, promoting the hydrolysis of GTP into GDP-bound Rab7, thereby facilitating the untethering of mitochondria-lysosome contact sites (Valm et al., 2017). Importantly, both TBC1D15 mutants (D397A or R400K) that lack GAP activity and Fis1 mutants (LA) that can recruit TBC1D15 to the mitochondria prevented untethering of the mitochondria-lysosome contact sites, giving rise to the prolonged duration of the contact sites (Sugiyama et al., 2008). Interestingly, TBC1D15 mutants do not impact the formation of contact sites, suggesting that TBC1D15 specifically regulates the untethering of contact sites rather than contact sites formation. Thus, it provides a new way to regulate the untethering process of mitochondria-lysosome contact sites. To sum up, mitochondrial TBC1D15/Fis1/lysosomal Rab7 signaling cascade is considered to be the key regulator in regulating the dynamic process of tethering and untethering at mitochondria-lysosome contacts sites (Yu et al., 2020).

## 4 The role of mitochondria-lysosome contact sites in regulating glucose uptake and cholesterol transport

Glucose uptake is very important for glucose metabolic homeostasis and glucose uptake in skeletal muscle and adipose tissues is regulated by GLUT4 membrane translocation, from intracellular vesicles to cell membranes (Leto and Saltiel, 2012). Membrane translocation of GLUT4 involves multiple Rab proteins (Wu et al., 2019). Rab7 is involved in membrane translocation of GLUT4 vesicles by endocytic trafficking or by the regulation of endosome translocation to lysosomes, or by mediating maturation of early endosome to late endosome or participating in sorting of early endosome (Girard et al., 2014). GLUT4 and Rab7 co-locate suggests that GLUT4 is also closely related to the late endosome pathway. Considering Rab7 is a key molecule regulating the tethering process of mitochondrial-lysosome contact sites, we speculate that Rab7 in endosomes and lysosomes may modulate GLUT4 translocation by regulating the formation of mitochondria-lysosome contact sites tethering. However, it is not known if Rab7 regulates GLUT4 translocation directly or indirectly through other specific molecules involved on mitochondria or lysosomes.

**FIGURE 3:**
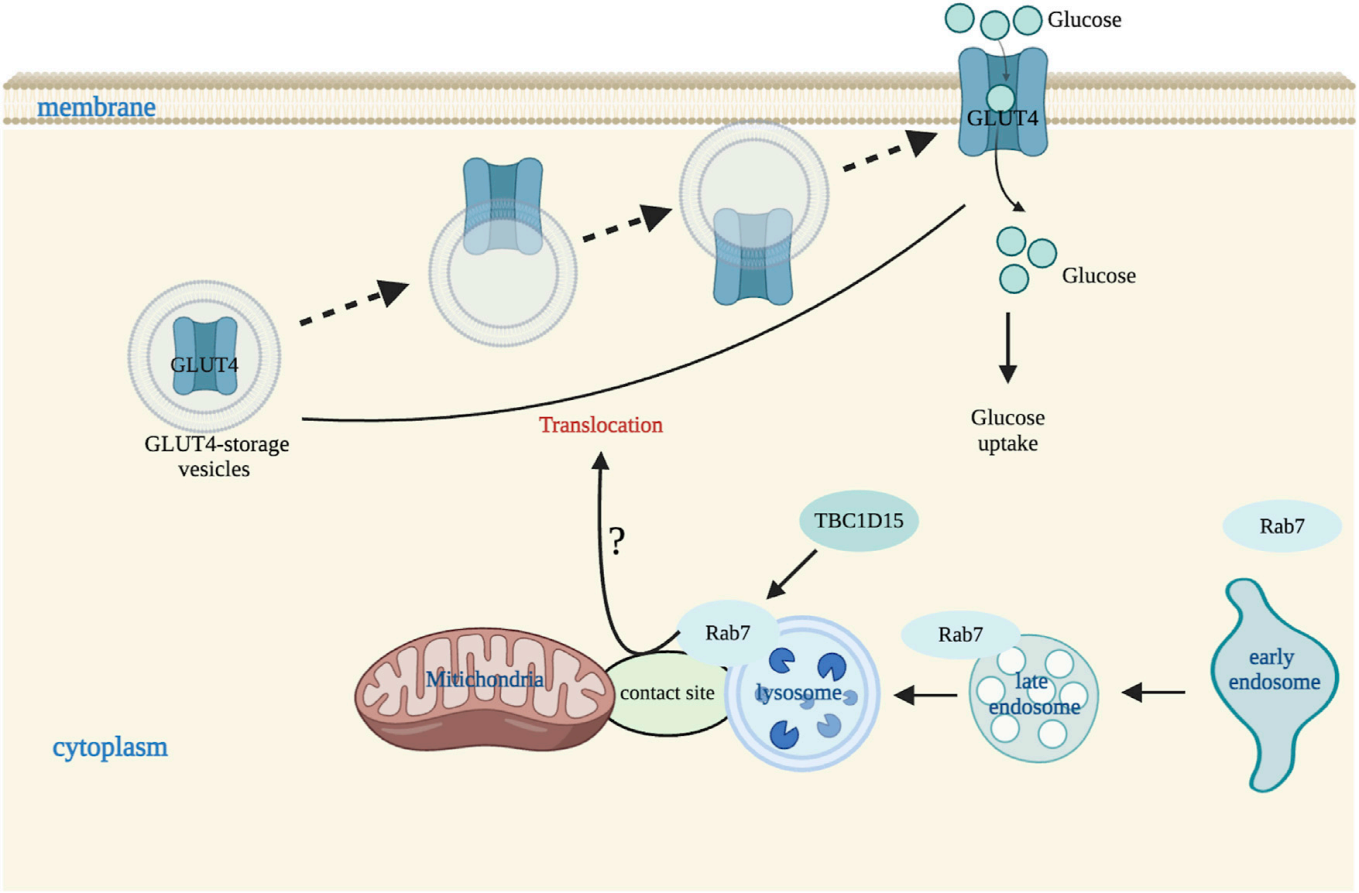
Mitochondria-lysosome contact sites regulate glucose transportation. In a physiological situation, Rab7 is involved in membrane translocation of GLUT4 vesicles possibly by endocytic trafficking or by the regulation of endosome translocation to lysosomes, or by mediating maturation of early endosome to late endosome or participating in sorting of early endosome. TBC1D15 also regulates GLUT4 vesicles membrane translocation by affecting Rab7 activity via endosomal late pathway. Image created with BioRender.com.

Besides, TBC1D15 can regulate the membrane translocation of GLUT4 vesicles through modulating Rab7 activity (Wu et al., 2019). Similarly, TBC1D15 untethers mitochondria-lysosome contact sites through its interaction with Fis1, activating the hydrolysis of Rab7 GTP, and subsequently modulating the morphology of both mitochondrial and lysosomes (Wong et al., 2019). Previous studies have found that loss of TBC1D15 resulted in abnormal mitochondria-lysosome contact sites observed in heart tissues from patients with ischemic cardiomyopathy (ICM), and from mouse hearts of ischemia/reperfusion (I/R) and neonatal mouse cardiomyocytes (NMCMs) exposed to hypoxia/reoxygenation (H/R), and this abnormality was improved by TBC1D15 overexpression. This suggests that TBC1D15 may promote the conversion among different pathways at mitochondria-lysosome interactions (Figure 3). However, if TBC1D15 regulates Rab7 activity through the mitochondria-lysosome contact sites to mediate the membrane translocation of GLUT4 vesicles remains to be further studied.

In addition to glucose transport and glucose uptake, mitochondria-lysosome contact sites also regulate cholesterol transfer (Henne, 2019). As cholesterol homeostasis is necessary to maintain lipid metabolism, it is important to explore the molecular mechanisms by which mitochondria-lysosome contact sites mediate the regulation of cholesterol metabolism between lysosomes and mitochondria. Previous studies have found that mTORC1 in the lysosome regulates cholesterol homeostasis through a variety of pathways. For example, it upregulated cholesterol biosynthesis via the ribosomal protein S6 kinase B1 (S6K1) or directly affected cholesterol trafficking in organelles by monitoring NPC1 (Dong et al., 2014; Düvel et al., 2010). NPC1 consists of three large luminal domains, namely, the N terminal domain (NTD), the middle luminal domain, and the cysteine-rich domain, and one transmembrane domain containing 13 transmembrane helices anchored to the lysosomal membrane (Qian et al., 2020). Among these, the NTD domain contains the transmembrane sterol-sensing domain (SSD) that binds to cholesterol (Lu et al., 2015), thereby regulating cholesterol output from the lysosome. If the SSD domain is lost or mutated, it may lead to local cholesterol accumulation (Vanier, 2015). In addition, mutation or deletion of NPC1 gene may cause lysosomal cholesterol storage disease (Du et al., 2015).

**FIGURE 4:**
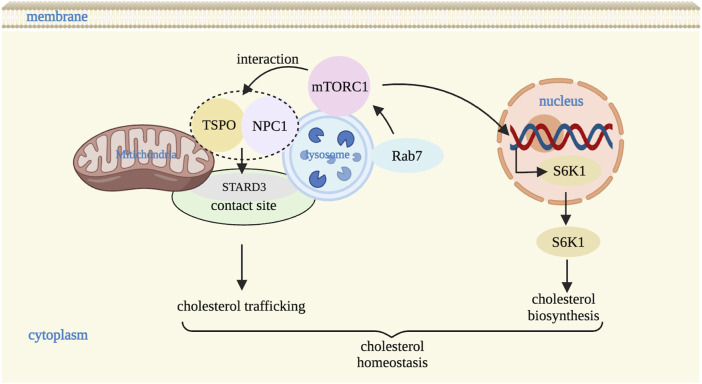
Mitochondria-lysosome contact sites regulate cholesterol homeostasis. mTORC1 in lysosome upregulates cholesterol biosynthesis via S6K1 and regulates the cholesterol trafficking between lysosomes and mitochondria via the interaction with NPC1 and TSPO through STARD3 (a cholesterol transporter) at contact site. mTORC1 activity is also regulated by Rab7. S6K1: S6 kinase B1. Image created with BioRender.com.

Several studies have found that Rag, Ras homolog enriched in brain (Rheb), Ras homolog (Rho), and Ras-associated protein (Rab) and a series of other lysosomal small GTPase are involved in mTORC1 regulating lysosome-mitochondria cholesterol trafficking in response to nutrients and stress (Lin et al., 2023; Zhu and Wang, 2020). Rab7, also facilitates the cargo trafficking among lysosomal associated inter-organelles (Guerra and Bucci, 2016). In Rab7 knockdown cells, the expression of mTORC1-associated proteins such as p-mTOR, p-S6K1, and phosphorylation levels of eukaryotic translation initiation factor 4E binding protein 1 (p-4EBP1) were significantly elevated suggesting that activity of mTORC1 is modulated by lysosomal Rab7 (Lin et al., 2023). In order to further confirm the relationship between Rab7 and mTORC1 in cholesterol transport from lysosomes to mitochondria, Lin et al. studied Rab7 activation in HepaRG and HepG2 cells and in liver of C57BL/6 mice, and found it was highly expressed, accompanied by decreased expression of mTORC1-related proteins, indicating that Rab7-mTORC1 signaling pathway may be a underlying mechanism for the cholesterol trafficking from the lysosome to mitochondria (Lin et al., 2023). Moreover, mTORC1 conveys cholesterol trafficking from lysosomes to mitochondria by regulating the interaction between NPC1 and TSPO, which further alleviated the accumulation of inter-organelle cholesterol through STARD3 (a cholesterol transporter) (Balboa et al., 2017; Charman et al., 2010) (Figure 4). And under conditions that increased the NPC1-TSPO interaction, cholesterol in both lysosomes and mitochondria was reduced, suggesting increased cholesterol transport elsewhere likely to the ER or plasma membrane through interaction with the pools of TSPO on the ER and plasma membrane respectively (Höglinger et al., 2019; Lin et al., 2023; Loth et al., 2020). Thus, NPC1 and TSPO are important molecular mechanisms involved in cholesterol transport between lysosome and mitochondrial contact sites (Lin et al., 2023). In addition, mTORC1 inhibition restores lysosomal proteolysis without correcting cholesterol storage, implicating aberrant mTORC1 as a pathogenic driver downstream of cholesterol accumulation (Davis et al., 2021). In short, mTORC1 is a key molecule that regulates cholesterol transport between lysosomes and mitochondria, and plays a very important role in lysosomal signaling pathway. However, the exact mechanism of mTORC1 regulating cholesterol transport through NPC1 needs further study.

## 5 Mis-regulation of mitochondria-lysosome contact sites in metabolic diseases

Many metabolic diseases have been closely associated with the function disorder of mitochondria and lysosomes, suggesting that the disruption of the crosstalk between these two organelles may be a latent molecular mechanism leading to metabolic disorders. Many studies recently have identified protein and functional links between various metabolic disorders and damaged dynamics and function at mitochondria-lysosome contact sites (Table 1).

**TABLE 1:** Proteins associated with metabolic diseases that have been related to mis-regulation of mitochondria-lysosome contact sites.

Metabolic disease	Description	Disease related protein	References
Insulin resistance	Peripheral tissue insulin sensitivity decreased	TBC1D15, Rab7, GLUT4	Zhang et al. (2005); Peralta et al. (2010); Wong et al. (2019); Wu et al. (2019)
Nonalcoholic fatty liver disease	Disrupted cholesterol homeostasis in liver	mTORC1, NPC1,	Davis et al. (2021); Lin et al. (2023); van den Boomen et al. (2020); Gosis et al. (2022); Lan et al. (2022)

Insulin resistance (IR) refers to the reduced sensitivity and reactivity of insulin target organs (liver, adipose tissue and skeletal muscle) to endogenous or exogenous insulin, resulting in lower glucose uptake and utilization than normal (Haas et al., 2016). IR is the main cause of many metabolic diseases and is also a closely related to T2DM (Schiavon et al., 2021). Therefore, understanding the molecular mechanism of lower glucose uptake and utilization is particularly important for the prevention and treatment of IR. Since skeletal muscle is the main place of glucose metabolism, skeletal muscle glucose metabolism is crucial for the maintenance of blood glucose balance in the whole body and the prevention of metabolic diseases (Poewe et al., 2017). At present, studies have found that glucose uptake and utilization in skeletal muscle is mainly an insulin-dependent mechanism, the specific process of which is that insulin acts on insulin receptors in skeletal muscle and then directly transduces signals to insulin receptor substrates such as insulin signaling pathway (INSR/IRS/PI3K/AKT). In this signaling pathway, activated AKT phosphorylates several proteins involved in glucose uptake. TBC1D4 is a direct downstream substrate of AKT, and activated TBC1D4 triggers the membrane translocation of GLUT4 and promotes glucose uptake (Chen et al., 2011).

The molecular mechanism of IR is not only related to the weakening of GLUT4 membrane translocation, but also closely correlated with mitochondrial damage and lysosome dysfunction (Cheng et al., 2020; Wu et al., 2019). TBC1D15 has been shown to be related to numerous cellular processes (Jongsma et al., 2020; Peralta et al., 2010; Takahara et al., 2014). It has been reported that TBC1D15 regulates the functions of mitochondria and lysosome (Cisneros et al., 2022; Yamano et al., 2014). Furthermore, TBC1D15 untether mitochondria-lysosome contact sites by activating Rab7 GTP hydrolysis through its interaction with Fis1. This process ultimately influences the morphology of mitochondria and lysosomes (Wong et al., 2019). Other studies have found that knockout of TBC1D15 resulted in dysfunction of mitochondria-lysosome contact sites, while its overexpression enhanced the conversion among diverse pathways of mitochondria and lysosome interacting, shifting from mitochondria-lysosome contact sites to mitophagy (Sun et al., 2022; Wu et al., 2019; Yu et al., 2020).

Moreover, it has been found that TBC1D15 knockout led to more GLUT4 translocation into late endosomes and lysosomes and its degradation through lysosome pathway, with Rab7 distribution becoming more concentrated within certain areas in cytoplasm. This ultimately reduces glucose uptake and contributes to IR (Wu et al., 2019). Notably, mitochondria-lysosome contact sites may act as a platform for metabolic interchange between these two organelles because communication between organelles usually occurs through membrane contact sites (Eisenberg-Bord et al., 2016; Phillips and Voeltz, 2016). Disturbance of these metabolic exchange processes can result in the extravagant accumulation of substances, enlargement of lysosome volume, and consequent lysosomal dysfunction.

Collectively, TBC1D15 plays an extremely important role in shaping the morphology and functions of mitochondria and lysosomes, as well as in regulating mitochondria-lysosome contact sites and the transport of cellular substance such as GLUT4 through the endosomal system. TBC1D15 acts as a critical molecule between mitochondria-lysosome contact sites and facilitates maintaining good mitochondrial quality control via restituting lysosomal acidification (Yu et al., 2020). Defects in TBC1D15 may be a latent factor driving IR in the pathogenesis of T2DM. It could represent a novel target for clinical treatments aiming to address IR. However, further in-depth research is necessary to fully understand the function of TBC1D15 in the other pathological situation, particularly with regards to IR.

Nonalcoholic fatty liver disease (NAFLD) is a another common chronic metabolic disease, with a global prevalence of 25%, and the prevalence is increasing (Powell et al., 2021). Its main clinicopathological feature is the excessive deposition of fat in hepatocytes in addition to alcohol and other definite liver damage factors (Ahmed et al., 2015). It is closely related to metabolic syndrome such as obesity and hyperlipidemia (Haas et al., 2016), therefore, contents of cholesterol and TG in NAFLD are often significantly increased (Heeren and Scheja, 2021). Many *in vitro* and *in vivo* experiments and clinical trials have confirmed that the disruption of cholesterol homeostasis greatly promotes the occurrence and development of NAFLD (Esquejo et al., 2018; Mouchiroud et al., 2019). Recent studies have emphasized that the communication between multiorganelles is lysosome centered to maintain intracellular cholesterol homeostasis (Henne, 2019) *via* facilitating inter-organelle cholesterol trafficking (Wong et al., 2019; Yañez and Leiva, 2022). This process is likely associated with the interaction of proteins related to cholesterol trafficking, such as mTORC1 and NPC1. Activation of mTORC1 signaling has been found to inhibit the interaction between lysosomal NPC1 and mitochondrial translocator protein (TSPO), resulting in the untethering of mitochondria-lysosome contact sites and cholesterol accumulation, indicating that mTORC1 serves as a central component of lysosomal signaling, mediating cholesterol trafficking from lysosomes to mitochondria by modulating the interaction between NPC1 and TSPO (Lin et al., 2023).

It is worth noting that the crucial role of Rab7 in lysosomal cholesterol export is dependent on NPC1 (van den Boomen et al., 2020). Moreover, downregulation of lysosomal Rab7 enhances disorders of cholesterol transport mediated by mTORC1 from lysosomes to mitochondria, indicating that the Rab7-mTORC1 signaling pathway in the lysosome regulates cholesterol transport from lysosomes to mitochondria, which in turn contributes to cholesterol-related lipotoxicity and liver damage (Lin et al., 2023). Furthermore, mitochondrial dysfunction is also a typical pathological characteristic of NAFLD (Park et al., 2018). Activating of the mTORC1 signaling pathway is closely connected with cholesterol-related lipotoxicity, which contributes to the progression of NAFLD (Lu et al., 2020). NPC1 knockout in HEK293T cells, overexpression of mTORC1 caused a significant increase in cholesterol aggregation in lysosomes, subsequently causing mitochondrial dysfunction. But mTORC1 inhibitor Torin1 significantly mitigated this phenotype change (Davis et al., 2021).

Finally, other major metabolic diseases, including hypertension, hyperlipidemia, and metabolic syndrome, have also been associated with pathological signaling pathway related in mitochondria and lysosomes. However, questions remain whether the exchange disturbance of inter-organelles at the mitochondria-lysosome contact sites is a pathogenesis of these diseases. Future studies will enhance our understanding of the precise mechanisms that may be related to the mitochondria-lysosome contact sites to various metabolic diseases.

It also remains to be determined whether damaged mitochondrial-lysosome contact sites in organs like the liver, fat tissue, skeletal muscle contribute directly to disease progression or as a consequence of other pathological processes. It is worth noting that many of the disease mutations impact the dynamics of lysosomes or mitochondria. Understanding the interaction between damaged organelles and mitochondria-lysosomal contact sites and their functions remains a principal task for further research in this area.

## 6 Conclusion and future perspectives

Mitochondria-lysosome contact sites have gained increasing recognition as central in metabolic processes associated with various tissues/organs and overall homeostasis. These contact sites are dynamically formed and play key roles in liver, skeletal muscle, and adipose tissue. Notably, multiple protein complexes can modulate the initial tethering and consequent untethering dynamics at mitochondria-lysosome contact sites. These contact sites have been shown to regulate multitude of cellular functions, such as mitochondrial lysosomal network dynamics, organelle motility, and metabolite transport between organelles. For example, TM4SF5-enriched mitochondria-lysosome contact sites modulate glucose catabolism by promoting cholesterol output of mitochondrial reprogramming (Kim et al., 2024). Importantly, dysfunction or mutation of the mitochondria-lysosome contact sites have been observed in models of different metabolic diseases such as IR, NAFLD, and LSD, suggesting that dysfunction of the mitochondria-lysosome contact sites may be an important cause of metabolic diseases.

Future research on the additional regulators of mitochondria-lysosome contact sites tethering and their exploration of new functions mediated by these contact sites may be indispensable for elucidating the role of this pathway in metabolic diseases, which will help understand the pathogenesis of these diseases and develop new therapies for treating relevant conditions.
