# Supplementary material for: A new perspective on the regulation of glucose and cholesterol transport by mitochondria-lysosome contact sites
Source: Front Physiol. 2024 Sep 3;15:1431030. doi: 10.3389/fphys.2024.1431030 (PMC11405319; doi:10.3389/fphys.2024.1431030)
Supplement: Supplementary file 1 [file DataSheet1.docx]

Supplementary Material

## 1. Supplementary Figures


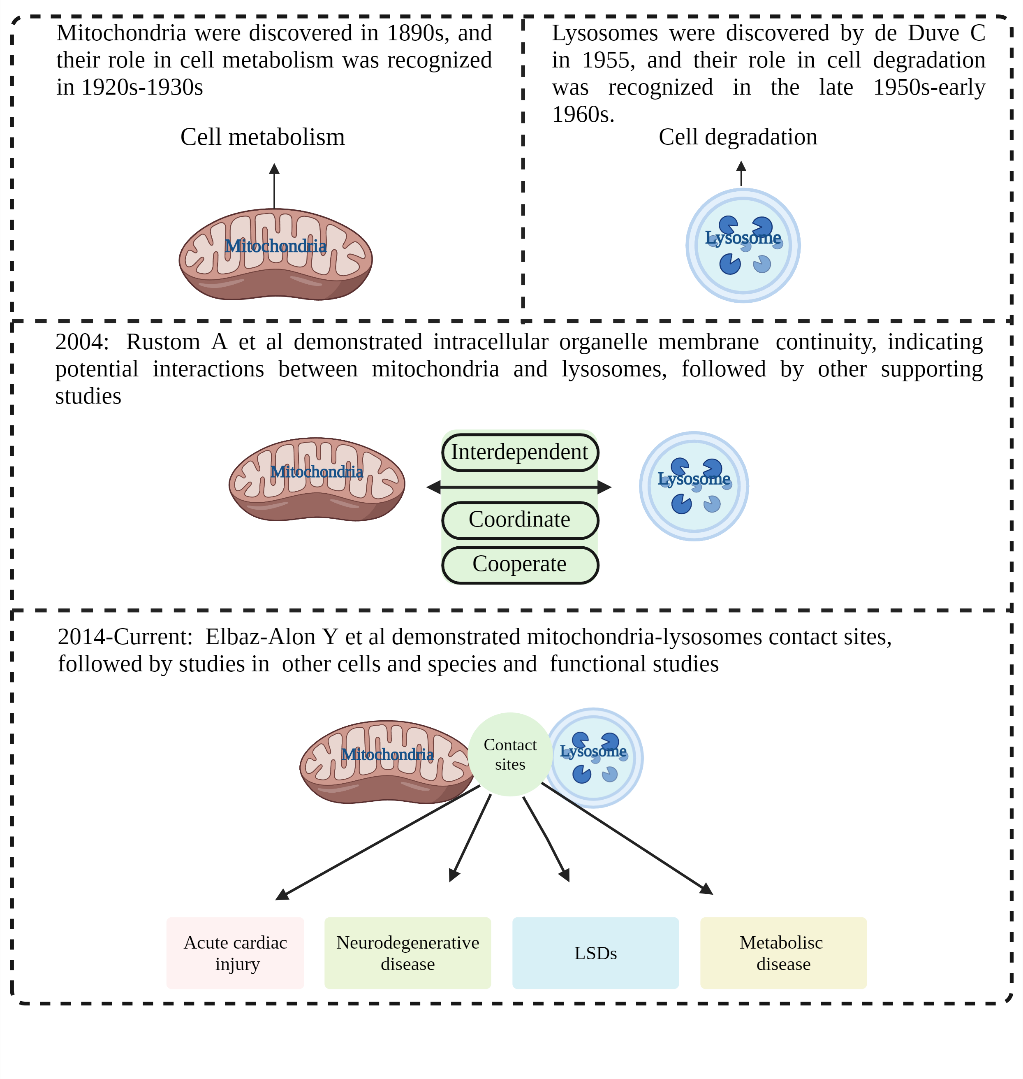


**Supplementary Figure 1.** Historical view of the interaction between mitochondria and lysosomes. LSDs: Lysosomal storage-disease. Image created with BioRender.com.

Figure 1. Historical view of the interaction between mitochondria and lysosomes. The early view was that mitochondria and lysosomes were independently responsible for cell metabolism and cell degradation respectively. The more recent view is that they are interdependent and coordinately to perform and regulate cell metabolism and signal transductions. The current view is that mitochondria and lysosomes form dynamic membrane contact sites, and abnormalities of these contact sites are associated with various diseases such as acute myocardial injury, neurodegenerative disease, LSDs and metabolism disease. LSDs: Lysosomal storage-disease. Image created with BioRender.com.


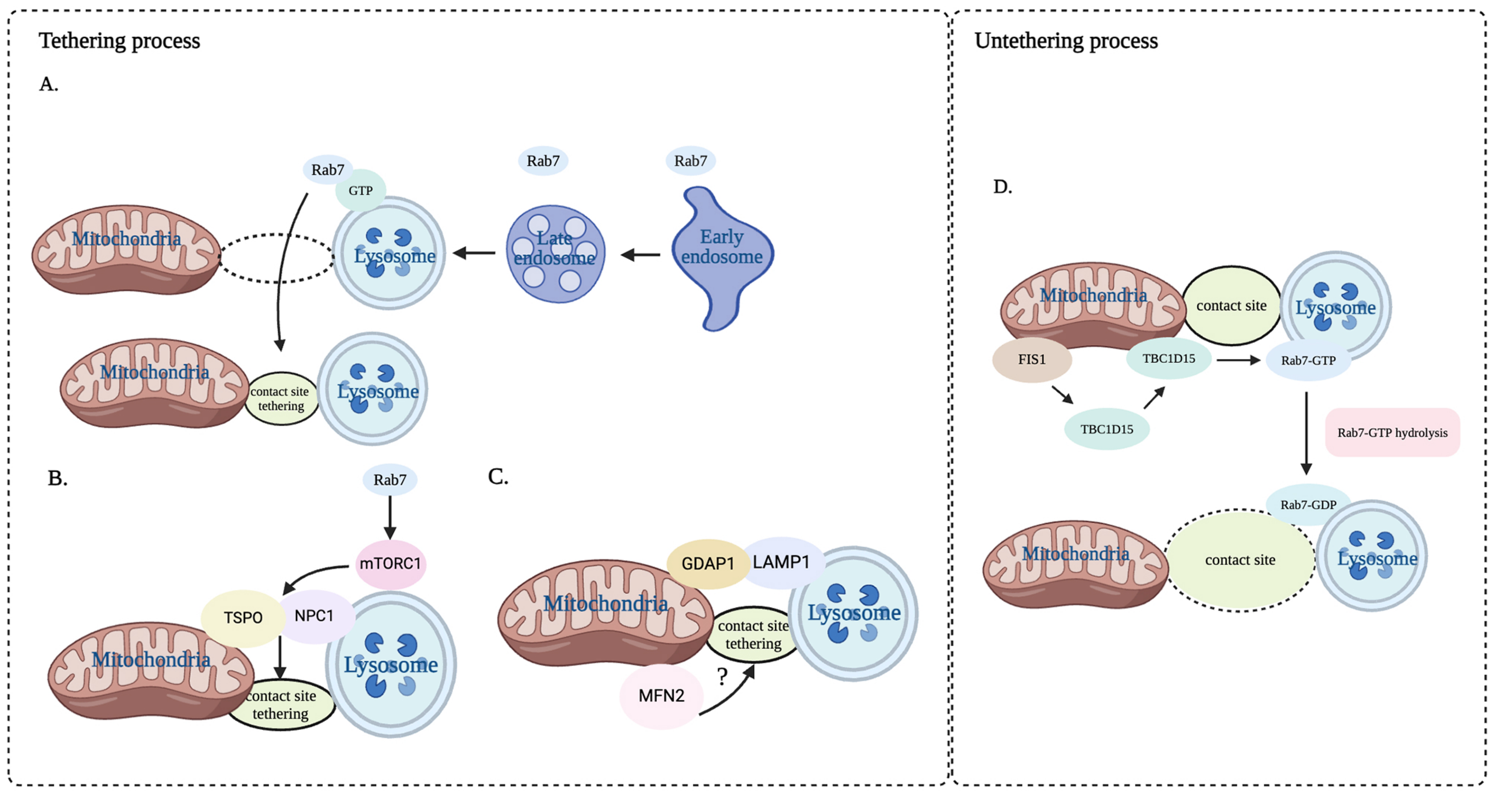


**Supplementary** **Figure 2.** Dynamic process of tethering and untethering at mitochondria-lysosome contact sites. This dynamic process is regulated by a variety of proteins that form the mitochondrial and lysosomal membranes. (A) Rab7 in the late endosome accesses the contact sites. (B) Rab7 regulates tethering by facilitating the interaction of TSPO and NPC1 through mTOR1. (C) GDAP1 interacting with LAMP1 enhances the tethering of mitochondria-lysosome contact site, and MFN2 is also involved in the regulation of mitochondria-lysosome contact sites. (D) Untethering process primarily regulated by Rab7 GTP hydrolysis driven by TBC1D15. NPC1: Niemann-Pick type C1. TSPO: translocator protein. MFN2: protein mitofusin. Image created with BioRender.com.


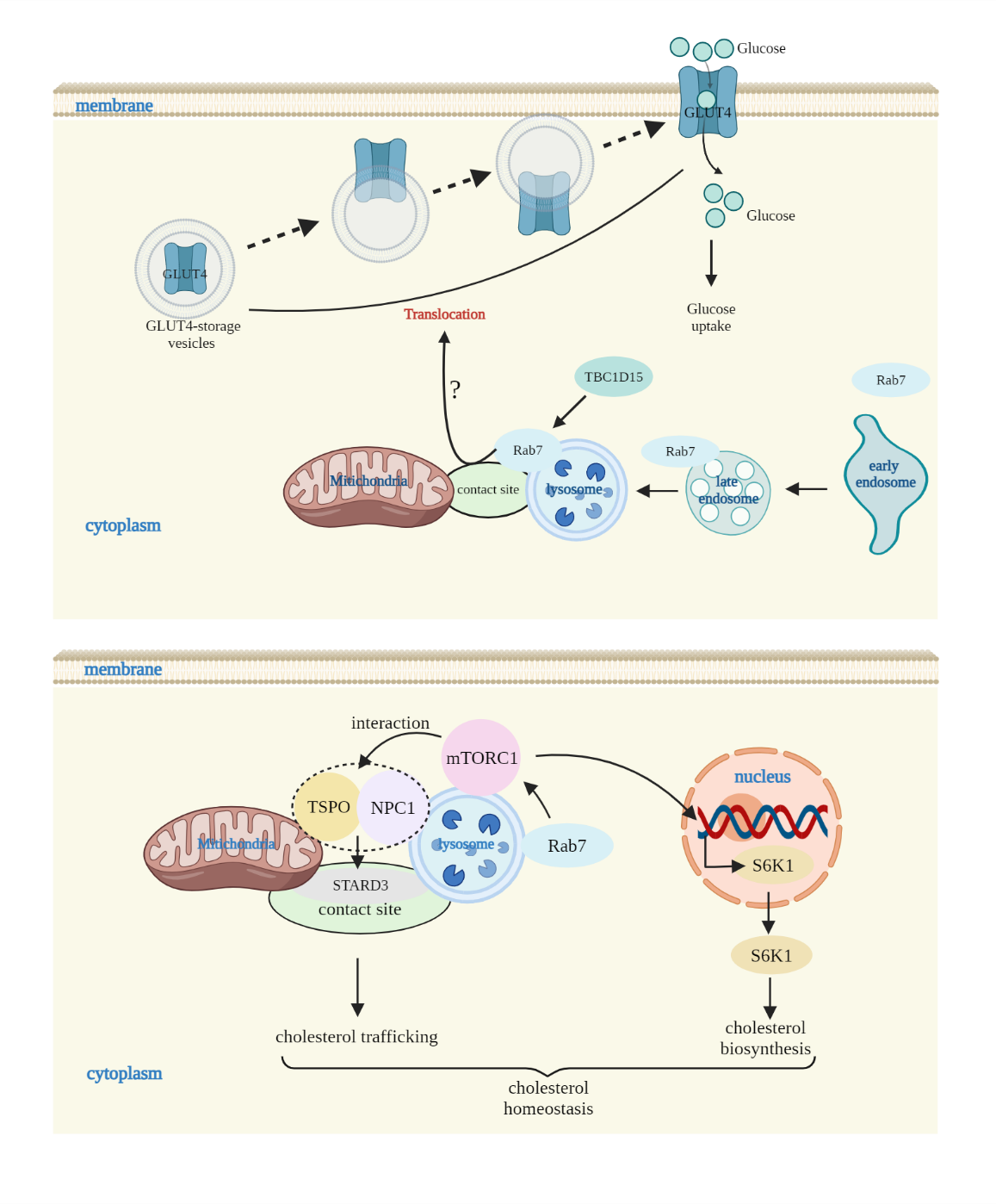


**Supplementary Figure 3.** Mitochondria-lysosome contact sites regulate glucose transportation. In a physiological situation, Rab7 is involved in membrane translocation of GLUT4 vesicles possibly by endocytic trafficking or by the regulation of endosome translocation to lysosomes, or by mediating maturation of early endosome to late endosome or participating in sorting of early endosome. TBC1D15 also regulates GLUT4 vesicles membrane translocation by affecting Rab7 activity via endosomal late pathway. Image created with BioRender.com.


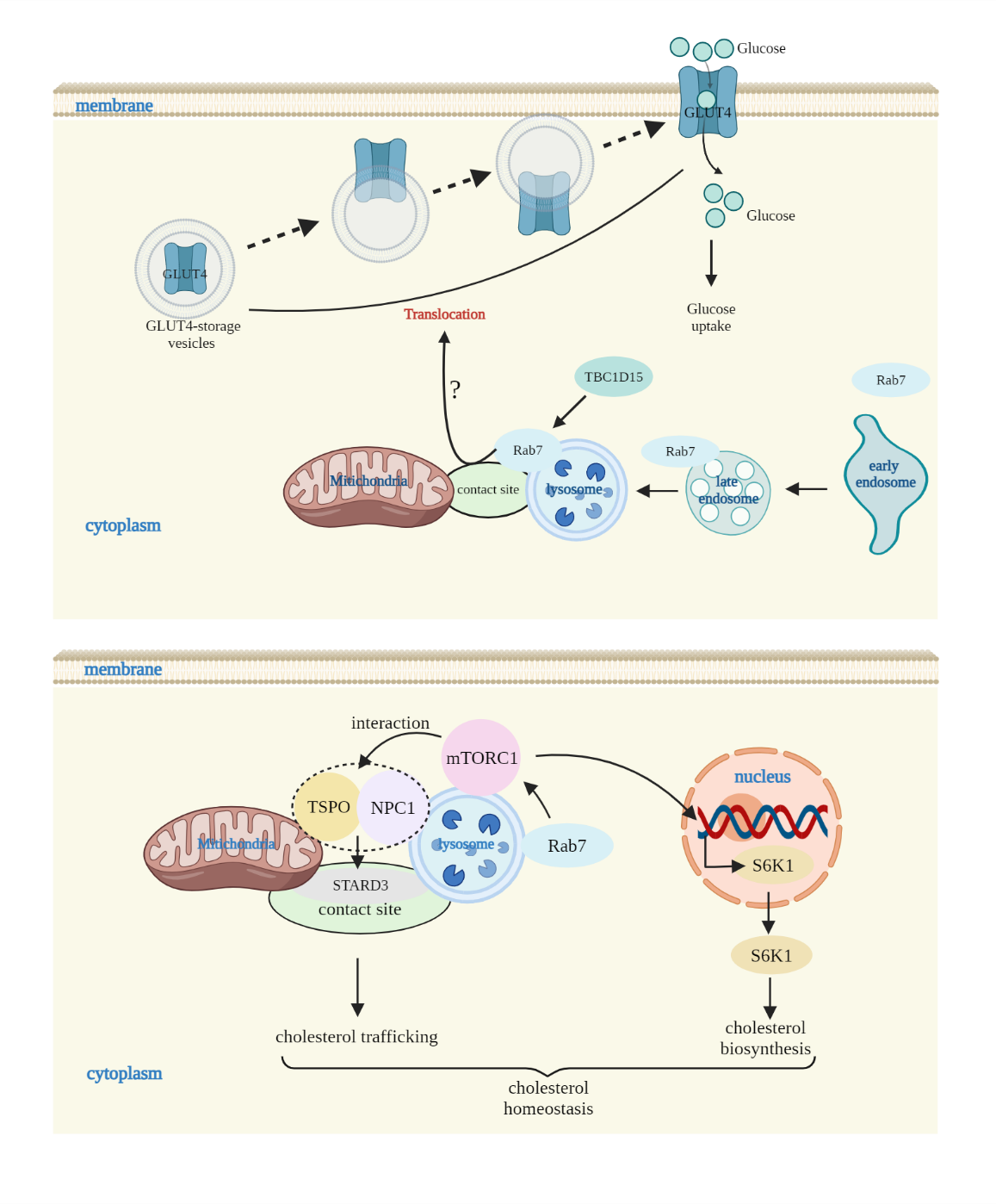


**Supplementary Figure 4.** Mitochondria-lysosome contact sites regulate cholesterol homeostasis. mTORC1 in lysosome upregulates cholesterol biosynthesis via S6K1 and regulates the cholesterol trafficking between lysosomes and mitochondria via the interaction with NPC1 and TSPO through STARD3 (a cholesterol transporter) at contact site. mTORC1 activity is also regulated by Rab7. S6K1: S6 kinase B1. Image created with BioRender.com.

**2. Supplementary Table**

**Supplementary Table 1.** Proteins associated with metabolic diseases that have been related to mis-regulation of mitochondria-lysosome contact sites

| Metabolic disease | Description | Disease related protein | References |
| --- | --- | --- | --- |
| Insulin resistance | Peripheral tissue insulin sensitivity decreased | TBC1D15, Rab7, GLUT4 | (Zhang et al., 2005; Peralta et al.,201  0; Wong et al., 2019;Wu et al., 2019) |
| Nonalcoholic fatty liver disease | Disrupted cholesterol homeostasis in liver | mTORC1, NPC1,  TSPO | (Davis et al., 2021; Lin et al., 2023; van den Boomen et al., 2020; Gosis et al., 2022; Lan et al., 2022) |
